# Response to the Ukrainian war: Support to the Ukrainian health professionals – war refugees

**DOI:** 10.1093/eurpub/ckac131.553

**Published:** 2022-10-25

**Authors:** A Petráková, N Veselá, M Ponomarenko, S Hrdličková, M Barták, M Dlouhý

**Affiliations:** 1 School of Public Health, Institute for Postgraduate Medical Education, Prague, Czechia; 2 Teaching, Institute for Postgraduate Medical Education, Prague, Czechia

## Abstract

**Issue/problem:**

There are currently more than 300,000 war refugees from Ukraine in the Czech Republic, including several hundred health workers of various health professions, including physicians, dentists, pharmacists, nurses and others. The Institute for Postgraduate Medical Education (IPVZ) on behalf of the Ministry of Health prepares and organizes the approbation exams of physicians, dentists and pharmacists who as foreign nationals have obtained medical qualification outside the EU and wish to practice their profession in the Czech Republic or the EU.

**Description of the problem:**

To follow up on new legislation of the Czech Republic, valid from 21 March 2022, focused on the Ukrainian war refugees and their stay in the Czech Republic, IPVZ immediately formulated its strategic response and support to an urgent needs of the Ukrainian refugees with a focus on health professionals and their integration to the Czech health system. IPVZ and its School of Public Health (SPH) work on capacities development courses, in particular on the Czech language courses and on introduction to the Czech health system.

**Results:**

First 3 Czech language courses, held on 2 May -1 July 2022, were completed by 34 attendees. Next 10 courses will start on 1 August 2022. There is an interest in both face-to-face and online Czech language courses. In parallel, we provide the introduction to the Czech health system through consistent creation of bilingual cycle of short videos, first videos are available free of charge on IPVZ web site at https://ua.ipvz.cz.

**Lessons:**

Interest in the Czech language classes indicates its importance for all Ukrainian health professionals - war refugees, who plan to stay in the Czech Republic and to work in the Czech health system in their health professions -physicians, nurses and others. Cycle of short bilingual videos is an important support tool for adoption of the Czech health system terminology and for their preparation for the approbation exams.

**Key messages:**

• Institute for Postgraduate Medical Education in Prague formulated its immediate strategic response to the Ukrainian war and needs of the Ukrainian war refugees with a focus on health workers.

• Intensive Czech language courses supplemented by the cycle of short bilingual videos, introducing the Czech health system, available free of charge at https://ua.ipvz.cz, fully support MoH CZ policy.

